# Ridge Preservation Compared With Spontaneous Healing in Extraction Sockets With Buccal Dehiscence: A Randomized Clinical Trial

**DOI:** 10.1111/jre.70097

**Published:** 2026-04-06

**Authors:** Cristina Valles, Pedro Diz‐Iglesias, Lory Abrahamian, Neus Carrió, Matteo Albertini, Jose Nart

**Affiliations:** ^1^ Department of Periodontology Universitat Internacional de Catalunya Barcelona Spain

**Keywords:** alveolar ridge preservation, bone regeneration, buccal dehiscence, dental implants, patient‐reported outcomes, postoperative pain, tooth extraction

## Abstract

Alveolar ridge preservation using a bone allograft and collagen membrane significantly limited hard and soft tissue dimensional changes and promoted buccal bone gain compared with spontaneous healing in sockets with buccal dehiscence defects. This approach reduced the need for additional bone grafting, despite higher short‐term postoperative morbidity.

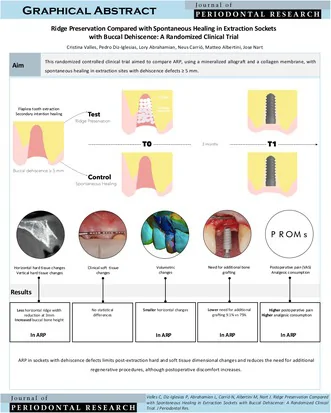

## 1 Introduction

Alveolar bone remodeling after tooth extraction is well documented, particularly in sites presenting with thin or damaged buccal bone walls [1, 2, 3]. Although alveolar ridge preservation (ARP) has demonstrated benefits in limiting physiological bone resorption [4], outcomes appear to be related to the integrity and thickness of the buccal bone wall prior to tooth extraction. Nonetheless, there is limited information concerning the effect of ARP on sockets with dehiscence defects [5, 6, 7]. This randomized controlled clinical trial aimed to compare ARP—using a mineralized allograft and a collagen membrane—with spontaneous healing in extraction sites with dehiscence defects ≥ 5 mm, assessing (1) radiographic hard tissue dimensional changes, (2) clinical and volumetric soft tissue changes, and (3) patient‐reported outcome measures.

## 2 Methods

The study was conducted at the Department of Periodontology, Universitat Internacional de Catalunya (UIC), Barcelona. The research protocol was approved by the Ethics Committee of UIC (Ref. PER‐ECL‐2016‐02), adhered to the principles of the Declaration of Helsinki, and was registered on ClinicalTrials.gov (NCT03548896).

Twenty‐six patients requiring extraction of a single tooth in the maxillary esthetic zone (second premolar to second premolar) with a buccal bone dehiscence ≥ 5 mm were enrolled between November 2016 and March 2021, and randomized 1:1 into two groups. Sample size calculation was based on total bucco‐lingual ridge width as the primary outcome. A ≥ 3‐mm intergroup difference was considered clinically relevant; assuming *α* = 0.05, *β* = 0.20, and a 20% dropout rate, a minimum of 12 patients per group was required.

Teeth were indicated for extraction due to non‐restorable caries, endodontic treatment failure, fracture, or prosthetic reasons. Eligibility was determined clinically immediately after extraction by using a periodontal probe at the mid‐buccal aspect, measuring the distance from the gingival margin to the buccal bone crest. CBCT imaging was subsequently used for baseline characterization and dimensional outcome assessment.

The test group received ARP using a mineralized cancellous allograft (Osteopure, European Cell and Tissue Bank) and a cross‐linked collagen membrane (*Ossix Plus*, *Datum Dental* Ltd.), while the control group was allowed to heal spontaneously. In both groups, all extractions were performed flapless, and healing occurred by secondary intention. Three months post‐extraction implant placement was performed. Bone‐level implants were inserted subcrestally (i.e., 1 mm) following the manufacturer's recommendations. Diameters of 3, 3.5, or 4.0 mm, and lengths of 10, 12, and 14 mm were used. Three‐dimensional positioning was standardized and performed following a prosthetically driven protocol under supervision of the same experienced clinician. When required, residual peri‐implant bone defects were treated with MCA and covered with the previously described collagen membrane.

Surgical procedures were carried out by postgraduate residents under continuous supervision of the same experienced periodontist (C.V.), following a standardized protocol. Three patients were excluded from the per‐protocol analysis (one patient did not receive the allocated intervention, and two patients did not attend the implant placement surgery at 3 months).

Cone‐beam computed tomography (CBCT) scans and standardized digital models (STL) were obtained at baseline and at 3 months, when implant placement was performed. Hard tissue outcomes included horizontal ridge width measured at 1, 3, and 5 mm below the crest, as well as buccal and palatal vertical height changes (Figure 1). Facial soft tissue levels and keratinized tissue width (KTW) were assessed using customized stents. STL superimpositions were used to quantify volumetric changes at the gingival margin and at 1, 3, and 5 mm apical to the margin. Postoperative pain and analgesic consumption during the first week were recorded using a visual analog scale (VAS). The need for additional bone grafting at the time of implant placement was also documented.

**FIGURE 1:**
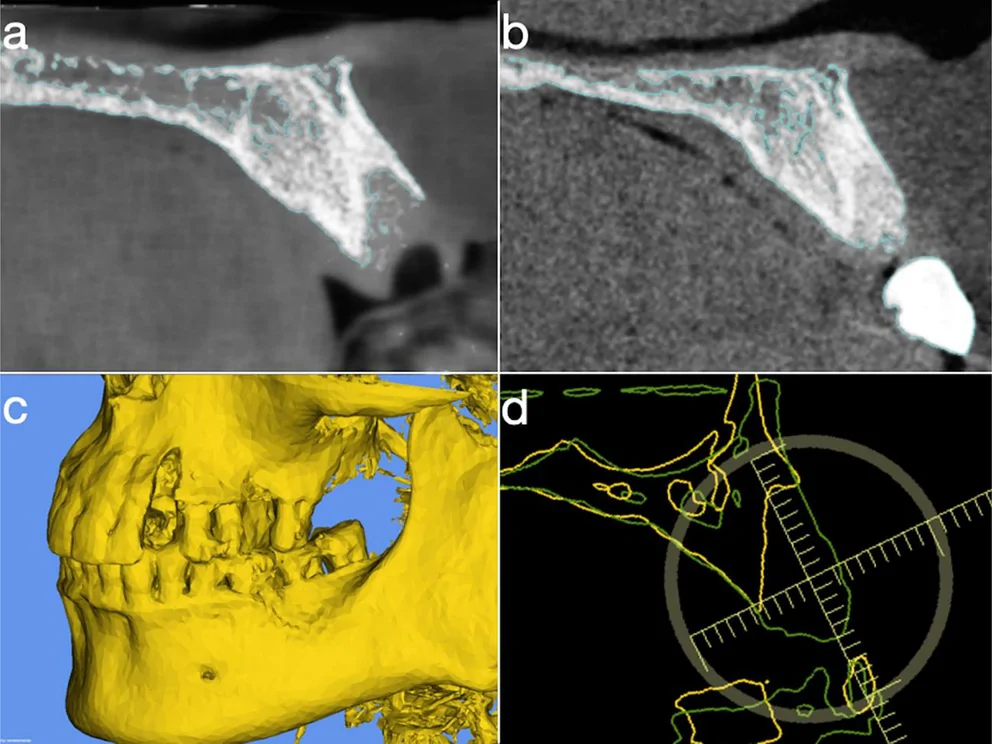
CBCT analysis using SMOP software. (a) surface extraction of CBCT at baseline; (b) surface extraction of CBCT at 3 months; (c) STL surface model generated by the software; (d) STL superimposition and linear measurements.

The study was powered to detect differences in total bucco‐lingual ridge width change at 3 months (primary outcome); all other variables were considered secondary outcome variables.

## 3 Results

After 3 months, the ARP group exhibited smaller horizontal ridge width reductions at all measured levels, reaching statistical significance at 3 mm (*p* = 0.014) and borderline significance at 5 mm (*p* = 0.056). Mean reductions at 1, 3, and 5 mm below the crest were −0.84 mm (SD 1.46), −0.35 mm (SD 0.69), and −0.49 mm (SD 1.45) in the ARP group, compared with −3.40 mm (SD 3.03), −3.02 mm (SD 2.46), and −2.40 mm (SD 2.06) in the control group. Buccal bone height increased significantly in the ARP group [5.50 mm (SD 3.02)] compared with the control group [1.79 mm (SD 2.99); *p* = 0.025], whereas no significant differences were observed for palatal height (Table 1).

**TABLE 1:** Hard‐ and soft‐tissue dimensional changes and patient‐reported outcomes in ARP and control groups from baseline to 3 months.

Variable	All (*n* = 23), mean ± SD	ARP (*n* = 11), mean ± SD	Control (*n* = 12), mean ± SD	*p*
**Radiographic changes**				
HW change at 1 mm (*n* = 21)	−2.06 ± 2.63	−0.84 ± 1.46	−3.40 ± 3.03	0.091
HW change at 3 mm (*n* = 23)	−1.74 ± 2.26	−0.35 ± 0.69	−3.02 ± 2.46	**0.014**
HW change at 5 mm (*n* = 23)	−1.49 ± 2.01	−0.49 ± 1.45	−2.40 ± 2.06	0.056
HB change (*n* = 20)	3.46 ± 3.45	5.50 ± 3.02	1.79 ± 2.99	**0.025**
HP change (*n* = 23)	−0.15 ± 1.44	0.32 ± 1.61	−0.57 ± 1.19	0.218
**Dimensional changes**				
Linear change at GM (*n* = 22)	−2.29 ± 1.22	−1.73 ± 0.99	−2.75 ± 1.24	**0.025**
Linear change at 1 mm (*n* = 22)	−2.32 ± 1.04	−1.76 ± 0.77	−2.78 ± 1.03	**0.035**
Linear change at 3 mm (*n* = 22)	−1.79 ± 1.02	−1.19 ± 0.76	−2.29 ± 0.95	**0.008**
Linear change at 5 mm (*n* = 22)	−1.57 ± 0.97	−0.93 ± 0.76	−2.09 ± 0.81	**0.004**
**Soft tissue level changes**				
Facial soft tissue level at T0 (mm)	−0.20 ± 1.30	0 ± 1	−0.38 ± 1.55	0.655
Facial soft tissue level at T1 (mm)	−0.30 ± 1.28	0.18 ± 1.19	−0.75 ± 1.23	0.122
Facial soft tissue level changes (mm)	−0.11 ± 1.23	0.18 ± 1.42	−0.38 ± 1.03	0.266
**Keratinized tissue changes**				
Keratinized tissue at T0 (mm)	4.93 ± 1.76	5.32 ± 1.79	4.58 ± 1.73	0.303
Keratinized tissue at T1 (mm)	3.83 ± 1.59	4.86 ± 1.34	2.88 ± 1.15	**0.003**
Keratinized tissue changes (mm)	−1.11 ± 1.85	−0.45 ± 1.68	−1.71 ± 1.85	0.190
**Pain/discomfort (VAS)**	4.00 ± 2.13	5.55 ± 1.63	2.58 ± 1.44	**0.001**
**Analgesic consumption (pills/week)**	9.65 ± 6.06	14.45 ± 1.81	5.25 ± 5.14	**< 0.001**

*Note:* Bold numbers are statistically significant, *p* < 0.05.

Abbreviations: ARP, alveolar ridge preservation; GM, gingival margin; HB, buccal ridge height; HP, palatal ridge height; HW, horizontal ridge width; SD, standard deviation.

Dimensional analysis showed significantly smaller horizontal changes in ARP‐treated sites at all evaluated levels. At the gingival margin, mean change was −1.73 mm (SD 0.99) in the ARP group compared with −2.75 mm (SD 1.24) in the control group; at 1, 3, and 5 mm apical to the margin, changes were −1.76 mm (SD 0.77), −1.19 mm (SD 0.76), and −0.93 mm (SD 0.76) in the ARP group compared with −2.78 mm (SD 1.03), −2.29 mm (SD 0.95), and −2.09 mm (SD 0.81) in controls, respectively (*p* < 0.05 for all comparisons).

KTW showed a greater mean reduction in the control group [−1.71 mm (SD 1.85)] than in the ARP group [−0.45 mm (SD 1.68)]; however, this difference did not reach statistical significance (*p* = 0.190). Additionally, ARP‐treated sites exhibited slight coronal migration of the soft tissue margin [0.18 mm (SD 1.42)], while control sites showed apical displacement [−0.38 mm (SD 1.03)]; nevertheless, the differences between groups were not statistically significant (*p* = 0.266).

Patient‐reported outcomes revealed significantly higher postoperative pain scores [5.55 (SD 1.63) vs. 2.58 (SD 1.44)] and analgesic consumption in the ARP group [14.45 pills/week (SD 1.81) vs. 5.25 (SD 5.14) pills/week; *p* < 0.001]. Despite increased postoperative morbidity, the need for additional bone grafting at implant placement was substantially lower in the ARP group (9.1%) than in the control group (75%).

## 4 Discussion

The present findings indicate that ARP effectively limits post‐extraction hard and soft tissue dimensional changes in extraction sockets with buccal dehiscence defects. In particular, ARP was associated with reduced horizontal ridge reduction and a significant gain in buccal vertical bone height, which may improve implant placement predictability and reduce the need for additional regenerative procedures [8].

The surgical protocol applied in this study (i.e., flapless approach with secondary intention healing) may have contributed to the favorable soft tissue outcomes observed, including the preservation of KTW. In addition, the slight coronal migration observed in the ARP group may be related to the maintenance of ridge volume in the presence of a grafted socket [9, 10].

Although implant placement was performed after a 3‐month healing period, previous histologic evidence has shown that ridge preservation using mineralized bone allograft may yield comparable amounts of new vital bone at 3 and 6 months of healing, supporting the chosen timing.

Several limitations should be acknowledged. First, all surgical procedures were performed by postgraduate students and operator experience may represent a limitation; however, standardized protocols and constant supervision by the same experienced clinician minimized the risk of differential impact between groups. Second, the limited sample size reduced the statistical power of the study, and the assumption that horizontal hard‐tissue differences below 3 mm are clinically irrelevant may have led to an underestimation of the effect of ARP. Finally, the short follow‐up period of 3 months restricts the interpretation of long‐term dimensional changes. Further studies with larger patient samples and extended follow‐up are needed to confirm these findings.

## 5 Conclusions

In extraction sockets with buccal dehiscence defects ≥ 5 mm, ARP using a mineralized allograft and a collagen membrane effectively limited post‐extraction hard and soft tissue dimensional changes and promoted buccal vertical bone gain. These outcomes improved conditions for implant placement and reduced the need for additional regenerative procedures. However, ARP was associated with increased postoperative discomfort and higher analgesic consumption.

## Funding

This work was supported by the Beca Proclinic research grant. Bone allografts and membranes were provided by Salugraft; implants were donated by Klockner.

## Conflicts of Interest

The authors declare no conflicts of interest.

## Data Availability

The data that support the findings of this study are available from the corresponding author upon reasonable request.
